# Clinical Remarks on the Bowel-Affections of Children in the Medical Wards of the Mercy Hospital

**Published:** 1862-08

**Authors:** N. S. Davis

**Affiliations:** Professor of Clinical Medicine, &c.


CHICAGO MEDICAL EXAMINER.

N. S. DAVIS, M.D., Editor.

VOL. III. AUGUST, 1862. NO. 8.

### Original Contributions.

ARTICLE XXIV.

### CLINICAL REMARKS ON THE BOWEL-AFFECTIONS OF CHILDREN IN THE MEDICAL WARDS OF THE MERCY HOSPITAL.

By N. S. DAVIS, M.D., Professor of Clinical Medicine, &c.

Gentlemen:—It is only a few days since, that I directed your attention to a case of diarrhœa, occuring as a sequel of rubeola. The frequent occurrence of that disease during the months of July and August, especially in all the more populous cities of our country, and its fatality among young children, renders no apology necessary for calling your attention to it again, in connection with the several little children before you. You see in all of them a striking similarity of physiognomy or external appearance of disease. There is the same sober, melancholy expression of countenance; the same pale, thin lips, sunken eyes, and blanched skin; the same small weak pulse, and general emaciation, in each. You see their limbs attenuated, the muscles soft and flabby, and the skin hanging in dusky wrinkles on the neck. I am informed that they have had diarrhœa about two weeks; the discharges being thin, like green or yellowish water, and occurring as often as six or eight times in the twenty-four hours, with vomiting occasionally after drinking freely. Their skin over the trunk of the body, and especially over the abdomen, is warmer and dryer than natural, while the extremities are cool. A spell of restlessness and crying or moaning a few minutes before a passage from the bowels indicates the existence of abdominal pains or gripings, though in many cases these are almost entirely absent. They have little or no appetite for anything but water, although the thirst is, in some cases, so great that they drink almost any bland liquid greedily.

The cases of irritation of the mucous membrane of the alimentary canal, or the *summer complaints of children*, as met with in general practice, may be arranged into three groups. The first group embraces those cases in which the patient is suddenly attacked with copious vomiting and purging of serous fluid, sometimes tinged with bile, and sometimes hardly staining the napkin.

Under the depleting influence of these evacuations the countenance becomes pale and contracted, the eyes sunken, the pulse small and frequent, the extremities cold and shrunken, the urine scanty or entirely suppressed, and the mind lethargic, with spells of great restlessness. In the more severe attacks, these effects follow so rapidly that collapse and death are reached in from six to twenty-four hours.

In most cases, however, after the first eight or ten hours, the discharges become less frequent and copious, the vomiting occurring only when drink is taken, and the passages from the bowels being less both in quantity and frequency.

Still very little nourishment is retained by the stomach, and the greater part of that little is hurried through the intestines without change. Consequently the little patient continues rapidly to emaciate and, unless counteracted by appropriate treatment, will usually reach the stage of fatal exhaustion in from one to three weeks. In the advanced stage of some of these cases, in addition to all the ordinary symptoms of exhaustion, there occurs constant vigilance or wakefulness, with rolling of the head, tossing of the hands, and sometimes moaning. These symptoms induce the parents and nurses to think the disease "has gone to the head." And I have known several cases of this kind in which the attending physician had been applying cold applications to the head, blisters behind the ears, and, in two cases, even leeches and a calomel purge, under the impression that the symptoms indicated the supervention of inflammatory action in the brain or its membranes. I need not remind you that in the cases alluded to, such opinions and practice are entirely erroneous; and that the symptoms described are the result, not of inflammatory action in the brain, but of a true anemic condition of that organ. It is well known that very excessive losses of blood will often produce the most distressing feelings in the head, accompanied by morbid vigilance or wakefulness, and sometimes slight delirium. This anemic condition of the brain, whether from hemorrhage or from exhausting serous discharges, may be distinguished from inflammation and active congestion, by close attention to the pupil of the eye, the corotid arteries, and the anterior fontanelle, if this still remains open. It is well known that cerebral inflammation produces contraction of the pupils, fulness and hardness of the corotids, and fulness of the fontanelle. It is not until the inflammation has terminated in effusion sufficient to produce compression, that the pupils become dilated. But the same amount of effusion that would dilate the pupils, would produce also stupor or coma, and still greater fulness of the fontanelle. Whereas in anemia of the brain, the dilated pupil and staring expression is accompanied by restless sleeplessness instead of coma, by softness of the corotids, and by a sunken or depressed fontanelle. Many of the cases included in what we have called the first group, after presenting the active symptoms of cholera morbus for the first few days, undergo a different change. The vomiting ceases, the discharges from the bowels remain frequent, but become small in quantity, and composed chiefly of mucus, often streaked with blood. Simultaneous with this change in the discharges, more or less febrile reaction comes on, causing the skin to become dry and warm, especially over the abdomen and trunk of the body; the pulse more full and quick, and indications of more frequent and severe pains in the abdomen. In a word, the symptoms become indicative of ileo-colitis or dysentery.

The second group of cases embraces all those (and they make a majority of all the bowel-affections of children during the hot months of summer), which commence with simple, thin, or serous evacuations from the bowels, without the intermixture of either blood or mucus, and without accompanying fever. The evacuations in the several cases constituting this group vary much in their color, consistence, and frequency. In some cases they are from the beginning so thin and colorless as to leave no more stain or residue on the linen than turbid water; and so large in quantity as to prostrate the patients very rapidly; causing the skin to become blanched and cool, the eyes sunken, the pulse small and weak, with all the indications of approaching collapse. In many instances the discharges of this character, after continuing long enough to induce a decided deficiency in the watery and saline constituents of the blood, and considerable prostration, become smaller in quantity, less frequent, and mixed with a little mucus. At the same time a moderate febrile reaction takes place, causing the child to become more fretful and the abdomen more hot. The color of the intestinal discharges varies much, being sometimes green, at others yellow, and again white. They vary much also in consistence and odor; being sometimes thin as water and almost as odorless, and at others only semi-fluid, frothy, and extremely offensive.

If the fœces are closely examined they will generally be found to contain more or less of the food or drink taken, which has passed through the alimentary canal with little or no change, and also numerous epithelial cells from the surface of the mucous membrane. The urine is generally scanty in proportion to the copiousness of the intestinal discharges. If the progress of the disease is not arrested by appropriate treatment, the patient continues steadily to loose flesh and strength, until the emaciation is as extreme as in the last stage of pulmonary tuberculosis, and the little sufferer sinks quietly into the arms of death, from simple exhaustion. In some cases, however, after the disease has continued three or four weeks, the patients partially recover. That is, the stomach and upper part of the alimentary canal appear to recover their functions; the child takes nourishment well, retains it, and regains a degree of cheerfulness. The discharges from the bowels, however, continue more frequent than natural, varying from two to six or eight times in the twenty-four hours. They are usually semi-fluid, of a light yellow or greyish color, often frothy, and always more or less offensive. They often contain curds of milk and other undigested particles of food. The urine is generally less in quantity than natural, and contains such an excess of phosphates and lithates as to give it a whitish or milky appearance soon after being voided. In this state the patient usually recovers a good appetite, often indeed, a morbid craving for food. But so large a part of what is taken, is either hurried through the bowels or only partially assimilated, that the emaciation continues and sometimes slowly increases. The abdomen becomes gradually more and more tumid, until after a few months it is greatly distended, presenting a strong contrast to the emaciated extremities. The distended abdomen is generally tympanitic, indicating a simple accumulation of gases, though sometimes hard tumors may be felt through the walls of the abdomen, consisting of enlarged mesenteric glands.

When disease assumes this form, it generally takes the name of tabes-mesenterica or marasmus, and may continue one or two years before ending in death or recovery.

The third class or group of cases of bowel-affections occurring in young children, during the summer months, are distinguished from the two preceding classes, by the presence of distinct febrile action at the commencement of the attack. With the first onset of vomiting, or purging, or both, the skin is found hot and dry; the lips parched; the pulse more firm and frequent; and the patient more fretful, and exhibiting more signs of pain. If vomiting exists in these cases, it is generally more a frequent wretching or straining to vomit, with only a slight discharge of thin mucus, sometimes colorless but often tinged yellow or green with bile. Of course, whatever food or drink is taken is promptly rejected.

The intestinal evacuations are generally frequent, accompanied by signs of pain, and almost always affording traces of mucus. When the disease is limited chiefly to the ileum and upper part of the colon, the discharge is mostly a thin serous fluid, tinged green or yellow and presenting only traces of mucus. When the lower part of the colon and rectum are the chief seat of disease, evacuations are almost wholly mucus, more or less streaked with blood, and accompanied by tenesmus and straining. Emaciation progresses rapidly, and there is more restlessness, accompanied by a fretful, peevish temper, than in the other class of cases.

*Causes.*—If you credit the statements of mothers and nurses, you will have no difficulty in determining the efficient cause of all these intestinal-affections of summer. In nineteen cases out of twenty, they will tell you, with the utmost confidence, that it is "the teeth;" meaning thereby, that the growth of the teeth causing them to press on the gums, is the direct cause of the irritation of the mucous membrane of the alimentary canal. And in the twentieth case, they will be equally confident that *worms* are the cause of all the mischief. So confidently and universally are these notions entertained, that hundreds of mothers, especially of the poorer classes, wholly neglect to seek medical aid for their little ones when attacked with diarrhœa, until it has run on two, three, or even four weeks, and they are reduced to skeletons, with the skin hanging in folds on their emaciated limbs and necks, and they are in a hopeless state of exhaustion.

If you ask the mother why she neglected the child so long, her ready and uniform reply is: "Oh! it was teething." And so she has been looking every day for some one or more of the teeth to come through the gums, to afford relief; and by way of doing *something*, she has very likely given one or more doses of castor oil or vermifuge.

This, however, strictly in accordance with the homœopathic principle, "similia similibus curantur," is nevertheless only calculated to hurry the patient a little faster towards the grave. If you suggest a doubt as to the part the teeth play in causing the disease, you will be assured that the gums are "swollen," and that the child is constantly "biting the fingers, or nipple, or whatever else is put into its mouth," and this, in their estimation, is abundant proof that the teeth cause all the difficulty. Now, gentlemen, I call your attention particularly to this subject of teething, because the observation of many years has satisfied me, that the popular errors in relation to it, cause the needless sacrifice of thousands of infants annually.

Not only the mother and the nurse, make it an excuse for neglecting to seek judicious medical advice, in the first and most curable stage of the diseases of infancy, but many physicians are in the habit of directly encouraging this neglect, by telling those who bring their children for advice, with the milder forms of diarrhœa, "that they are *teething*, and it cannot be expected that they will be very well until that process is completed." But is it true, that the natural growth of the teeth and their exit from the gums, is a cause of disease? I think not. First, because it is a strictly physiological process, as much so, as the growth of the hair or nails. Second, because the gum is a structure neither endowed with a high degree of sensibility, nor supplied with nerves of extensive sympathetic relations. Third, because the constant disposition of the child to put everything in its mouth, and bite freely with its gums, proves them to be neither tender nor sensitive, and without these there can be no irritation.

That the growth of the teeth is not the cause of cholera infantum and the diarrhœas of summer, will be fully evident to you from the following well established facts:—

The teeth of children are growing at all seasons of the year, in January as much as in July; and hence, whatever diseases arise therefrom, would be liable to occur as much at one season as another. But the diseases under consideration are restricted in their prevalence almost entirely to the hottest part of the year. This, in our latitude, is embraced in the months of July, August, and September. So true is this, that the number of deaths from these diseases alone, gives to the months named, an average annual mortality more than double that of any other months in the year. And yet, notwithstanding these obvious and universally admitted facts, we daily hear mothers, nurses, and doctors talking of "teething" as the common and efficient cause of the intestinal complaints of summer.

Again, you will often hear it alleged that the eating of unripe and spoiled fruit and vegetables, is the principal cause of intestinal summer complaints. The fallacy of this is sufficiently shown by the fact, that a majority of the children attacked, are so young that they have not eaten fruit or vegetables of any kind.

The real causes of the summer complaints of children, both predisposing and exciting, will be easily inferred from a careful study of the localities, the season of the year, and the special circumstances under which they occur. They occur much more severely in densely populated cities than in rural districts. They are particularly prevalent in all those cities and populous towns of our country occupying that climatic belt or zone characterized by the greatest contrast of the seasons, that is, the greatest difference between the coldest days of winter and the hottest days of summer. This belt or zone, so far as regards that part of the United States east of the Rocky Mountains, is included between the paralells of 31° and 42° north latitude. In a large part of this territory the difference in temperature between the coldest days of winter and the hottest of summer, is, from 75° to 140° of Fahrenheit.

It can hardly be doubted, but this extreme annual variation of temperature operates as a predisposing cause of intestinal fluxes during the warm part of the year. This predisposition is also increased by habitual dampness of the atmosphere, by the various impurities that exist in the air of large cities, and by that morbid material or agent usually called malaria. Age, too, exerts an important predisposing influence; the attacks being far more numerous in infancy and early childhood, than at any subsequent period of life.

There are satisfactory anatomical reasons for this. The mucous membrane of the digestive organs at birth, is extremely delicate, and both the epithelial layer and the numerous glandular structures belonging to it, are imperfectly developed; so much so as to be fitted for contact with only the most simple fluids, such as good milk. These structures do not complete the development and acquire the compactness and diminished sensitiveness of complete or mature organization until the child is at least three years old.

But the period during which the most rapid development takes place, and during which there is consequently the greatest susceptibility to morbid impressions, is the first two years of life.

The teeth being a part of the digestive apparatus, their growth and protrusion through the gums, constituting what is familiarly called "teething," takes place during this same two years, and simply affords a visible index of the rapid development of the mucous membrane, and all other parts of that apparatus, by which it acquires that diminished sensibility, that increased compactness of structure, and its glandular appendages that secretory power, which enables it, not only to receive and digest a greater variety of food, but also, to withstand a greater variety and intensity of morbid impressions without inducing disease. Remember then, gentlemen, that the growth of the teeth is only a *coincident* of corresponding growth or development in the whole digestive canal; and to attribute the important diseases of the mucous membrane of that canal, during the first two years of life, to such growth of the teeth, is as absurd as it would be to attribute them to the *coincident* growth of the hair on the child's head, or the nails on its fingers.

While we attribute to the extremes of the seasons, confinement in the nursery, dampness of the atmosphere, malarious and other impurities, a predisposing influence; I am fully satisfied that the immediate exciting or efficient cause, is a high atmospheric temperature.

During thirteen years residence in this city, I have annually noted the commencement of the prevalence of diarrhœas and cholera affections among the children, and have uniformly found such commencement to be coincident with the first week of continuous hot summer weather. One or two hot days at a time is not sufficient; but when the heat is continuous for five or six in succession, it has been invariably accompanied and followed by more or less prevalence of diarrhœa and cholera infantum. This first week of hot weather in this locality, varies in the time of its occurrence in different years, but may be confidently looked for between the twentieth of June and the fifteenth of July. From that time to the end of July new attacks continue to multiply with rapidity. During the month of August new attacks are much less frequent, but a large proportion of the milder cases commencing in July, are not brought to the notice of the physician until August. So to, a very large proportion of the fatal cases that commence during the last half of July do not reach the fatal termination until in August. Hence, the month of August uniformly shows much the highest ratio of mortality, and is of course regarded by the people as the most sickly month of the year. But a careful observation and record in relation to the time of attack, will show that three-fourths of all the cases of simple diarrhœa or summer complaint, cholera infantum, and cholera morbus, date their commencement during the first three weeks of hot weather, which is almost uniformly in July. To understand the *modus operandi* of caloric, or a high temperature in producing disease, we must have a clear conception of the normal properties of living, organized matter, and the manner in which those properties may be modified by exterior agents. A careful analysis of the phenomena connected with organization and life, shows that every organized living structure, whether vegetable or animal, is possessed of two properties elementary and essential to the existence of matter in an organized and living state. The first, is an affinity by which the organic atoms are made to assume a definite arrangement, constituting the primary structures and types of organization. This property for convenience we call *vital affinity.*

The second, is a susceptibility to impressions from exterior influences, or a capability of being acted upon. This susceptibility must not be confounded with nervous *sensibility*, which is merely one of the functions of nerve structure, and not an elementary property of organized matter. The two elementary properties here alluded to, will be most clearly appreciated by reference to the simpler types of organization, such as the germinal cells of the ovarium, the egg, or the acorn. You examine the latter, for instance, and you will find the organic atoms of which it is composed, arranged in a certain, definite, and uniform manner, in strict obedience to a special *affinity*. That the particles thus arranged possess a special *susceptibility* is easily demonstrated by the action of certain exterior agents upon them.

Thus, the acorn lying dry upon the table, manifests no change any more than a piece of chalk lying by its side; but let a certain degree of caloric or heat and moisture be brought to bear upon it, and a change immediately commences, which, continued under the guidance of the special affinity among its particles, soon results in the development of a miniature oak. But the piece of chalk exposed to the same influences is only a piece of chalk still; thus demonstrating that the acorn, though passive or dormant, possessed a susceptibility peculiar to organized matter.

If you thus, see clearly what we mean by *vital affinity* and *susceptibility*, as the elementary properties of all organized living matter; you are prepared to understand the effects of a high temperature, both as a predisposing and exciting cause of disease. Caloric is one of those inponderable agents capable of pervading all matter, whether organic or inorganic; and its effect is to expand all bodies by causing the atoms of which they are composed to be separated farther from each other. Hence, it is the great antagonistic power to affinity whether simple, elective, or vital. Its direct effects upon the living tissues of the human system, constitute no exception to the general law of its operation upon other matter. Every successive addition of caloric or increase of temperature increases the expansion of the tissues, and of course lessens, in the same proportion, the vital affinity between the atoms of which the tissues are composed. If you wish for proof of this you have only to immerse your finger a few minutes in water of as low temperature as can be borne without injury; fit a ring accurately to it, and then immerse it the same length of time in water as warm as can be borne, and you will find the size of the finger so increased that you will probably be unable to get the same ring on or off. But caloric not only expands living tissues, thereby diminishing vital affinity, but it also *increases* their susceptibility. It is chiefly by its power, thus to diminish the tonicity or compactness of the tissues, while it increases their irritability or susceptibility, that caloric or a high temperature becomes an efficient predisposing or exciting cause of disease. Acting more directly on the cutaneous surface, and both by continuity of structure and close physiological sympathy, also on the whole internal mucous surface, a high atmospheric temperature renders these structures morbidly sensitive, while their expansion renders them more lax, and thereby puts them in the most favorable condition for sudden and rapid fluxes of fluids into and through them. Hence it is, that in the midst of summer all classes and all ages of people are more or less disposed to intestinal-affections, characterized by increased flow of fluids, such as diarrhœa, cholera morbus, etc. What is thus a predisposition in all classes, becomes, in children at that age when the mucous membrane is yet undergoing development, as already described, sufficient to constitute positive disease. If to this is added further depressing and relaxing effects of confinement in the nursery, or in the impure air of narrow streets and alleys, inhabited by the poor, and the smaller quantity of oxygen inhaled when the atmosphere is rarified by a high temperature, we shall have no difficulty in explaining the annually destructive prevalence of bowel-affections in young children, during the hot months of July and August.

But, gentlemen, I have unconsciously spent so much time in the present explanatory digression, and in the preceding examination of these children, that all comments on the pathology of the diseases under consideration, and on their treatment, must be deferred until the next clinic hour.

[*Continued in the next Number.*]

